# Effect of IL-34 on T helper 17 cell proliferation and IL-17 secretion by peripheral blood mononuclear cells from rheumatoid arthritis patients

**DOI:** 10.1038/s41598-020-79312-z

**Published:** 2020-12-17

**Authors:** Xin Li, Yimeng Lei, Ziyu Gao, Bei Zhang, Liping Xia, Jing Lu, Hui Shen

**Affiliations:** 1 Department of Rheumatology, 1st Affiliated Hospital of China Medical University, Shenyang, 110001 China; 2 Department of Rheumatology, 1st Affiliated Hospital of Jinzhou Medical University, Jinzhou, 121000 China; 3 104k class 86, China Medical University, Shenyang, 110001 China

**Keywords:** Rheumatoid arthritis, Preclinical research

## Abstract

Interleukin (IL)-34 is a new pro-inflammatory cytokine with elevated expression in rheumatoid arthritis (RA) patients. Our previous study showed that the frequency of T helper 17 (Th17) cells was also elevated in RA patients. Our study aimed to determine the effects of IL-34 on the proliferation, transcription factor expression and cytokine secretion of different subgroups of CD4 + T cells [Th1, Th2, Th17 and regulatory T (Treg) cells] in RA patients. Peripheral blood mononuclear cells (PBMCs) were isolated from the peripheral blood of 10 RA patients and stimulated with different concentrations of recombinant human (rh) IL-34 (0, 25, 50 and 100 ng/ml). Flow cytometry was used to determine the frequencies of the 4 subgroups of CD4 + T cells. Reverse transcription-PCR, western blotting and enzyme-linked immunosorbent assays were used to determine the mRNA and protein expression levels of transcription factors and cytokines. As a result, the frequency of Th17 cells was obviously increased under IL-34 stimulation. Moreover, the expression of the transcription factor retinoic acid-related orphan receptor (ROR-γt) and secretion of IL-17 by PBMCs were increased by stimulation with IL-34. However, there were no effects of IL-34 on transcription factors or cytokine secretion in Th1, Th2 and Treg cells. In conclusion, IL-34 can improve the proliferation of Th17 cells and expression of IL-17 in RA patients.

## Introduction

Rheumatoid arthritis (RA) is an autoimmune disease characterized by symmetrical synovitis in multiple joints^1^. Without regular treatment, joint deformity and disability occur in RA patients^2^. Although the pathogenesis of RA is not very clear, increasing evidence demonstrates that cytokines are the most important mediators of the inflammatory response in RA^3^, including tumour necrosis factor-α (TNF-α), interleukin (IL)-6 and IL-17. However, other cytokines, such as IL-4, IL-10 and IL-27, can help to control the inflammatory response in RA.

IL-34 is one of the newest members of the interleukin family^4^. Like macrophage-colony stimulating factor (M-CSF), IL-34 is a ligand of colony stimulating factor-1 receptor (CSF-1R)^3^. IL-34 has been found in many organs and tissues of the human body, including the heart, liver, spleen, lungs, kidneys, brain, and lymph nodes^3^. IL-34 exerts pleiotropic biological activities in specific tissues and cells, such as myeloid cells, epithelial cells, endothelial cells, fibroblasts, neurons, and cancer cells^5^. In pathological conditions, IL-34 seems to be related to many diseases ranging from inflammation to autoimmunity^6,7^. Previous studies have shown an association between IL-34 expression and RA severity^5^. IL-34 is able to upregulate IL-6 expression on fibroblast-like synoviocytes (FLSs) in RA patients^8^. Relatively high serum levels of IL-34 in RA patients correlate with a variety of RA features and are considered an independent risk factor for radiographic progression in RA^9–11^. Our previous study showed elevated levels of IL-34 in RA patients, which were associated with disease activity^11^.

CD4 + T cells can differentiate into a variety of effector subsets, including T helper type 1 (Th1) cells, Th2 cells, Th17 cells and regulatory T (Treg) cells. Th17 cells have been shown to have important functions in RA, and our previous study showed that the percentage of Th17 cells was obviously higher in RA patients than in healthy people^12^. Transcription factors play critical roles in specifying and maintaining the function of CD4 + T cells indistinct effector lineages^13^. T-box (T-bet) and GATA-binding protein 3 (GATA-3) are required for the differentiation of Th1 and Th2 cells, respectively. The transcription factor retinoic acid-related orphan receptor (ROR-γt) is essential for Th17 cell differentiation^14^. Moreover, forkhead box protein 3 (Foxp3) is a master regulator of Treg cells.

However, whether IL-34 affects the proliferation, transcription factor expression and cytokine expression of different subsets of CD4 + T cells in RA is still not clear. In this study, we evaluated the effects of IL-34 on Th1, Th2, Th17 and Treg cell proliferation and function to further elucidate the pathogenesis of IL-34 in RA.

## Materials and methods

### Preparation, stimulation and culture of peripheral blood mononuclear cells (PBMCs)

PBMCs were isolated from the peripheral blood of 10 newly diagnosed RA patients by centrifugation (20 °C at 1500 r/min for 10 min) using Lymphoprep (Fresenius KabiNorge AS, Norway). All patients fulfilled the 2010 American College of Rheumatology (ACR) criteria for RA^15^. The mean age of the RA patients (male/female: 4/6) was 54 (54.00 ± 5.03) years, and the mean disease duration was 6.17 (6.17 ± 3.89) years. None of the patients had any other diseases, and no disease-modifying antirheumatic drugs (DMARDs) were administered. All RA patients were in the acute active phase when enrolled. The present study was approved by the ethics committee of The First Affiliated Hospital of Jinzhou Medical University according to the Declaration of Helsinki. Written informed consent was provided by all the patients. All experiments were performed in accordance with relevant guidelines and regulations, including any relevant details.

PBMCs were suspended in 1640 medium (HyClone, USA) supplemented with 10% foetal bovine serum (FBS; CLARK, USA) and 2% penicillin and streptomycin. The PBMCs were cultured in 24-well culture plates at a density of 1.5*10^5^/ml/well. Anti-CD3 (0.5 μg/ml, R&D Systems, USA) and anti-CD28 (1 μg/ml, Abcam, England) antibodies were added to stimulate T lymphocyte activation for Western blot (3 days) and reverse transcription-PCR (3 days before RNA extraction) analyses. In addition, recombinant human (rh) IL-34 (0, 25, 50, and 100 ng/ml, R&D Systems) was added to the medium.

### Flow cytometric quantification of the percentages of Th1, Th2,Th17 and Treg cells

PBMCs were resuspended in culture medium in 24-well flat-bottomed plates at 2*10^6^ cells/well in 0.5 ml of medium and then stimulated with phorbol myristate acetate (PMA, 50 ng/ml) and ionomycin (500 ng/ml) for 4 h at 37 °C, with Golgi Plug (1 μg/10^6^ cells, Sigma-Aldrich) added for the last hour. For surface marker staining, cells were incubated with APCCy7-labelled anti-human CD4 (A161A1, BioLegend, USA) and PE-labelled anti-human CD25 (M-A251BD, Bioscience, USA) antibodies in a final volume of 100 μL of PBS for 15 min at room temperature protected from light. For intracellular staining, cells were washed, fixed, and permeabilized using Fix and Perm cell permeabilization reagents (BD Biosciences, USA). Intracellular cytokines were stained with BV421-labelled anti-IFN-γ (B27, BD Biosciences, USA), PE-labelled anti-IL-4 (8D4-8, BD Biosciences, USA) and AF647-labelled anti-IL-17A (N49-653, BD Biosciences, USA) antibodies for 30 min at 4 °C in the dark. For intranuclear staining, cells were washed, fixed, and permeabilized using Fix and Perm cell permeabilization reagents (BioLegend, USA). Intranuclear cytokines were stained with BV421-labelled anti-IL-10 (JES3-9D7, BD Biosciences, USA) and AF647-labelled anti-Foxp3 (259D/C7, BD Biosciences, USA) antibodies in a final volume of 100 μL of permeabilization solution for 60 min at room temperature in the dark. The percentages of Th cells and Treg cells among PBMCs was detected using a FACS Verse flow cytometer (BD Biosciences, San Jose, CA) and analysed with Flow Jo software version 7.6.1 (Tree Star, Ashland, USA). Dead cells and doublets were excluded from the analysis with live/dead cell dyes.

### Western blot analysis of the expression of T-bet, GATA-3, ROR-γt and Foxp3

Total protein was extracted from PBMCs on ice using RIPA lysis buffer (R0010, Solarbio, Beijing) according to the manufacturer’s protocol. The isolated protein was obtained after centrifugation at 14,000×*g* for 10 min. Fifty micrograms of total protein was separated by SDS-PAGE and transferred to polyvinylidene fluoride (PVDF) membranes (00010, Millipore Co., USA). After being blocked with 5% BSA for 2 h, the membranes were incubated with primary antibodies overnight at 4 °C on a shaker. The following primary antibodies were used: anti-RORγt (562197, BD, USA, 1:500), anti-T-bet (561262, BD, USA, 1:250), anti-GATA-3 (ab106625, Abcam, England, 1:1000) and anti-Foxp3 (ab20034, Abcam, England, 1:250). Then, the membranes were incubated with a secondary horseradish peroxidase-conjugated goat anti-rabbit (ab205718, Abcam, England, 1:1000) or anti-mouse (554002, BD, USA, 1:1000) antibody for 2 h. After the membranes were washed with TBST, chemiluminescence (ECL), a gel imaging apparatus (Bio-Rad, ChemiDoc MP, USA) and analysis software (Image Lab Software) were used.

### Reverse transcription-PCR analysis of the gene expression of TBX21, GATA-3, IL-17A, RORC, IL-10 and Foxp3

Total RNA was extracted from cultured PBMCs with TRIzol and examined by evaluating the A260/A280 ratio for values between 1.8 and 2.0. Reverse transcription of the total mRNA was performed using a routine method. A 0.5-μg sample of total RNA was reverse transcribed using the PrimeScript RT Master Kit. The resulting cDNA was used for amplification by RT-PCR with the SYBR Premix Ex TaqTM Kit and ABI Prism 7000 (Applied Biosystems, Norwalk, CT). The cDNA was subjected to 40 cycles of PCR amplification. Each cycle included 30 s of denaturation at 95 °C, 30 s of annealing at 60 °C, and 30 s of extension at 72 °C. β-actin was amplified as an internal control. The primers used in this study are listed in Table 1. The different gene expression levels were calculated using the 2^−ΔΔCt^ method, as previously described^16^.

**Table 1:** List of the sequence of gene primers.

Gene name	Forward (5′–3′)	Reverse (5′–3′)
*β-actin*	CATGTACGTTGCTATCCAGGC	CTCCTTAATGTCACGCACGAT
*TBX21*	CCGTGACTGCCTACCAGAATG-3	AACAGGATACTGGTTGGGTAGGA
*GATA3*	CGAGATGGCACGGGACACTA	TGGTCTGACAGTTCGCACAGG
*IL-17A*	CTCTGTGATCTGGGAGGCAAA	CTCTTGCTGGATGGGGACA
*RORC*	GCCAGAATGACCAGATTGTGCTT	AAGGCACTTAGGGAGTGGGAGA
*IL-10*	GAGATGCCTTCAGCAGAGTGAAGA	AGTTCACATGCGCCTTGATGTC
*Foxp3*	CTGGCAAATGGTGTCTGCAAGT	CTGCCCTTCTCATCCAGAAGATG

### Enzyme-linked immunosorbent assays (ELISAs) for IFN-γ*, *IL-4, IL-17A and IL-10

Cell culture supernatants were collected and stored in centrifuge tubes at -20 °C. The levels of IFN-γ, IL-4, IL-17A and IL-10 were measured with ELISA kits according to the manufacturer’s protocol (R&D, USA).

### Statistical analysis

Data with a normal distribution are shown as the mean ± standard error of the mean (SEM). Data with a non-normal distribution are shown as the median (IQR). GraphPad Prism 6.0 was used for statistical analysis. Statistical comparisons between four groups with normal distributions were performed by ANOVA. Statistical comparisons between four groups with non-normal distributions were performed with the Kruskal–Wallis test. Differences were considered statistically significant at values of *P* < 0.05.

## Results

### Effect of IL-34 on Th17 proliferation in RA patients

**Figure 1:**
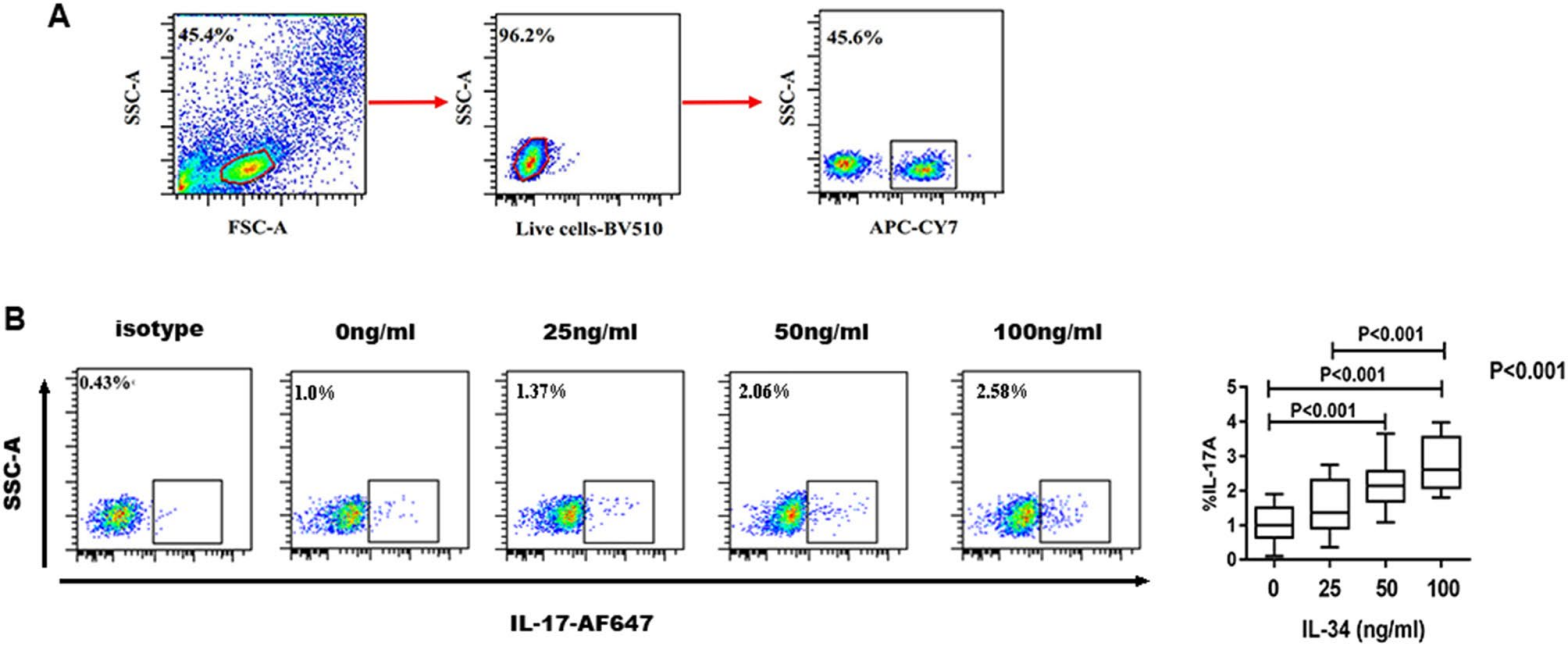
The frequency of Th17 cells stimulated with different concentrations of IL-34 by flow cytometry quantification. (**A**) The gating strategy for Th17 cells in PBMCs from the RA patient. (**B**) Pseudo color plots show representative flow cytometric data of CD4 + IL-17 + T cells. Bar charts show the frequency of Th17 cell. The differences between four groups were tested by ANOVA and after post Tukey test. PBMCs were stimulated with phorbol myristate acetate and ionomycin for 4 h with Golgi Plug added for the last hour before flow cytometry assay.

The effects of IL-34 on Th17 cell proliferation were detected by flow cytometry. PBMCs were isolated from RA patients and treated with different concentrations of rhIL-34 (0, 25, 50, and 100 ng/ml). IL-17 + T cells were gated in the CD4 + lymphocyte population (Fig. 1A). The frequencies of Th17 cells (IL-17 + CD4 + T cells) stimulated with different concentrations of rhIL-34 (0, 25, 50, and 100 ng/ml) were 1.028 ± 0.127%, 1.524 ± 0.188%, 2.145 ± 0.162%, and 2.751 ± 0.186%, respectively. There were statistically significant differences between 0 ng/ml and 50 ng/ml (*P* < 0.001), 0 ng/ml and 100 ng/ml (*P* < 0.001), and 25 ng/ml and 100 ng/ml (*P* < 0.001) (Fig. 1B). This shows that IL-34 can promote the proliferation of Th17 cells in a dose-dependent manner.

However, we did not find the same effect of IL-34 on cells from healthy controls (Supplementary Fig. 1A,B). These data suggest that the effect of IL-34 may be specific to RA.

### Effects of IL-34 on Th1, Th2 and Treg cell proliferation in RA patients

**Figure 2:**
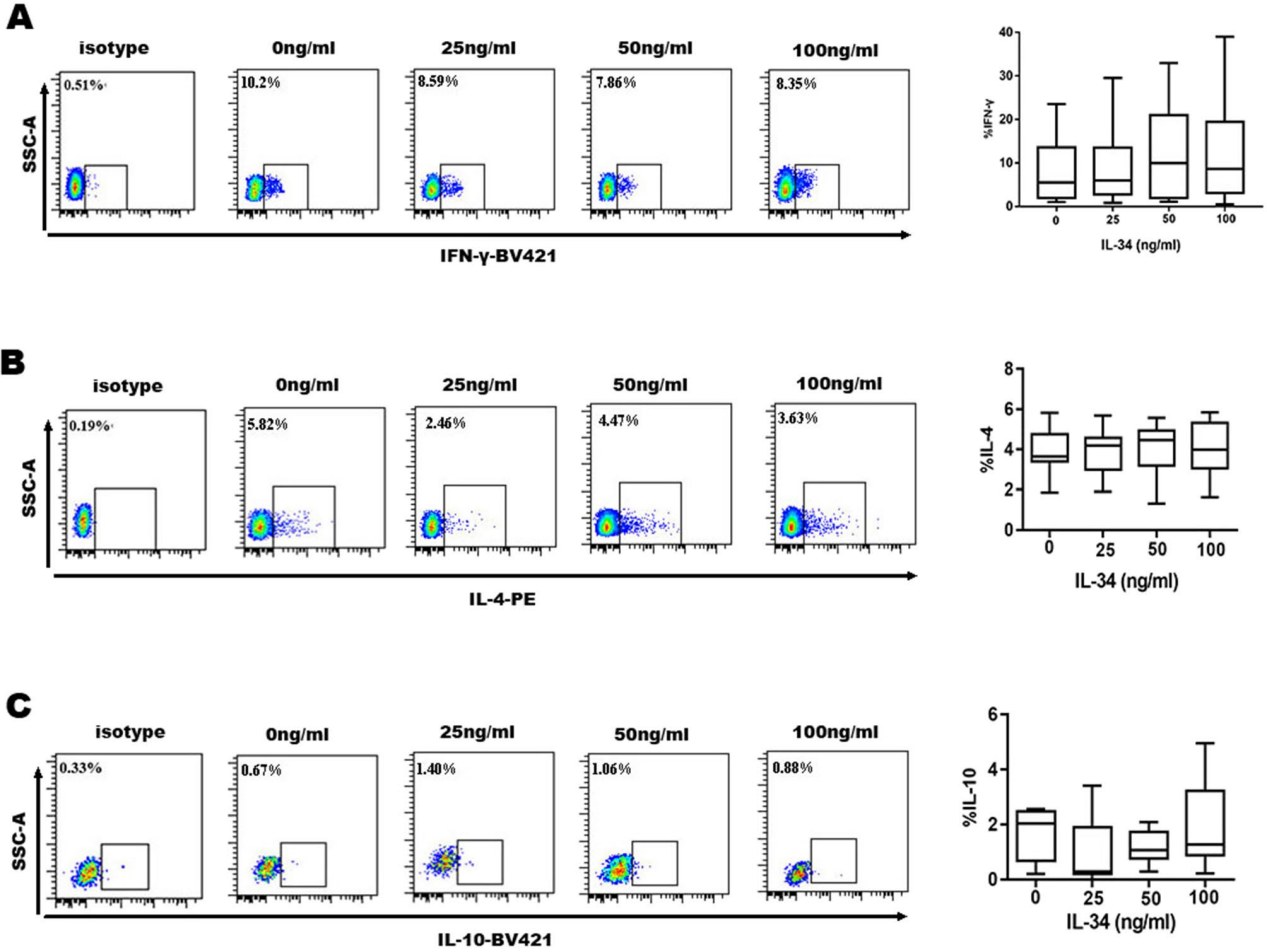
The frequency of Th1 cells, Th2 cells and Treg cells stimulated with different concentrations of IL-34 by flow cytometry quantification. (**A**) Representative flow cytometry plots for the frequency of CD4 + IFN-γ + Th1; Box & whiskers charts representing the frequency of Th1 cells with no significant difference in different concentrations of IL-34. (**B**) Representative flow cytometry plots for the frequency of CD4 + IL4 + Th2; Box & whiskers charts representing the frequency of Th2 cells with no significant difference in different concentrations of IL-34. (**C**) Representative flow cytometry plots for the frequency of CD4 + IL10 + Treg; Box & whiskers charts representing the frequency of Treg cells with no significant difference in different concentrations of IL-34. PBMCs were stimulated with phorbol myristate acetate and ionomycin for 4 h with Golgi Plug added for the last hour before flow cytometry assay. The differences between four groups were tested by Kruskal–Wallis.

The frequencies of Th1 cells (IFN-γ + CD4 + T cells) in the context of stimulation with different concentrations of rhIL-34 (0, 25, 50, and 100 ng/ml) were 3.65% (IQR = 1.49), 4.27% (IQR = 1.99), 12.44 ± 2.747% and 4.33% (IQR = 2.29), respectively. There were no significant differences among the groups (Fig. 2A).

The frequencies of Th2 cells (IL-4 + CD4 + T cells) in these four groups were 4.607 ± 0.797%, 4.694 ± 0.806%, 4.046 ± 0.351% and 8.61% (IQR = 16.38), respectively. There were also no differences among these frequencies (Fig. 2B). Moreover, the percentages of Treg cells were not significantly altered by the different concentrations of rhIL-34 (1.533 ± 0.380%, 0.29% (IQR = 1.99), 1.231 ± 0.243% and 1.817 ± 0.639%, respectively) (Fig. 2C).

### IL-34 stimulated the protein expression of ROR-γt in PBMCs

**Figure 3:**
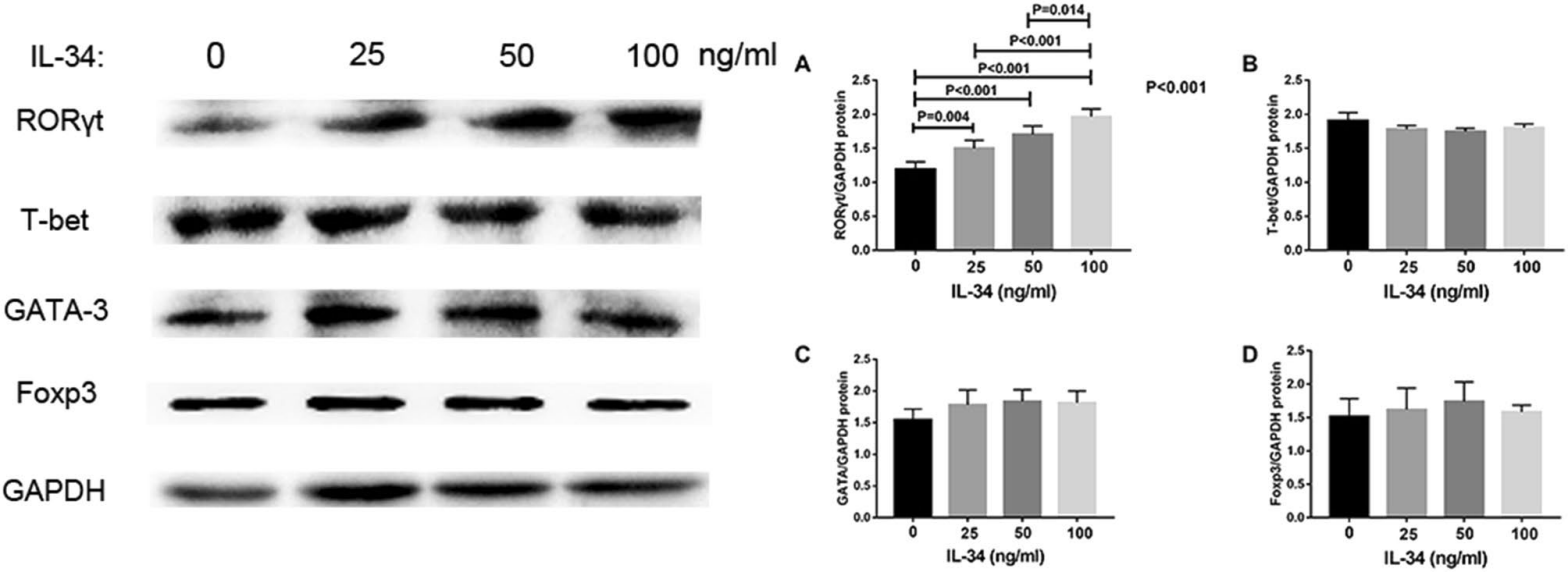
The difference between the protein expression of RORγt (**A**), T-bet (**B**), GATA3 (**C**), and Foxp3 (**D**) stimulated with different concentrations of IL-34 and anti-CD3 and anti-CD28 for 3 days by Western blot analysis. The differences between four groups were tested by ANOVA and after post Tukey test.

To further investigate the role of rhIL-34 inTh17 cells, we detected the effect of IL-34 on the expression of the transcription factor for Th17 cell differentiation (ROR-γt) by western blotting. The values of the ROR-γt/GAPDH ratio in the context of stimulation with rhIL-34 (0, 25, 50, or 100 ng/ml) were 1.192 ± 0.0489, 1.499 ± 0.0529, 1.704 ± 0.0567, and 1.965 ± 0.051, respectively. There were significant differences between the 0 ng/ml group and 25 ng/ml group (*P* = 0.004), 0 ng/ml group and 50 ng/ml group (*P* < 0.001), 0 ng/ml group and 100 ng/ml group (*P* < 0.001), 25 ng/ml group and 100 ng/ml group (*P* < 0.001), 50 ng/ml group and 100 ng/ml group (*P* = 0.014) (Fig. 3A). This shows that IL-34 may promote the expression of ROR-γt in a dose-dependent manner.

We also detected the effect of IL-34 on the expression of the transcription factors for Th1 cells (T-bet), Th2 cells (GATA-3) and Treg cells (Foxp3) by western blotting. However, there were no significant differences identified for any of these three transcription factors among the different concentrations of rhIL-34 (Fig. 3B–D).

### IL-34 upregulated the mRNA expression of RORC and IL-17 in PBMCs

**Figure 4:**
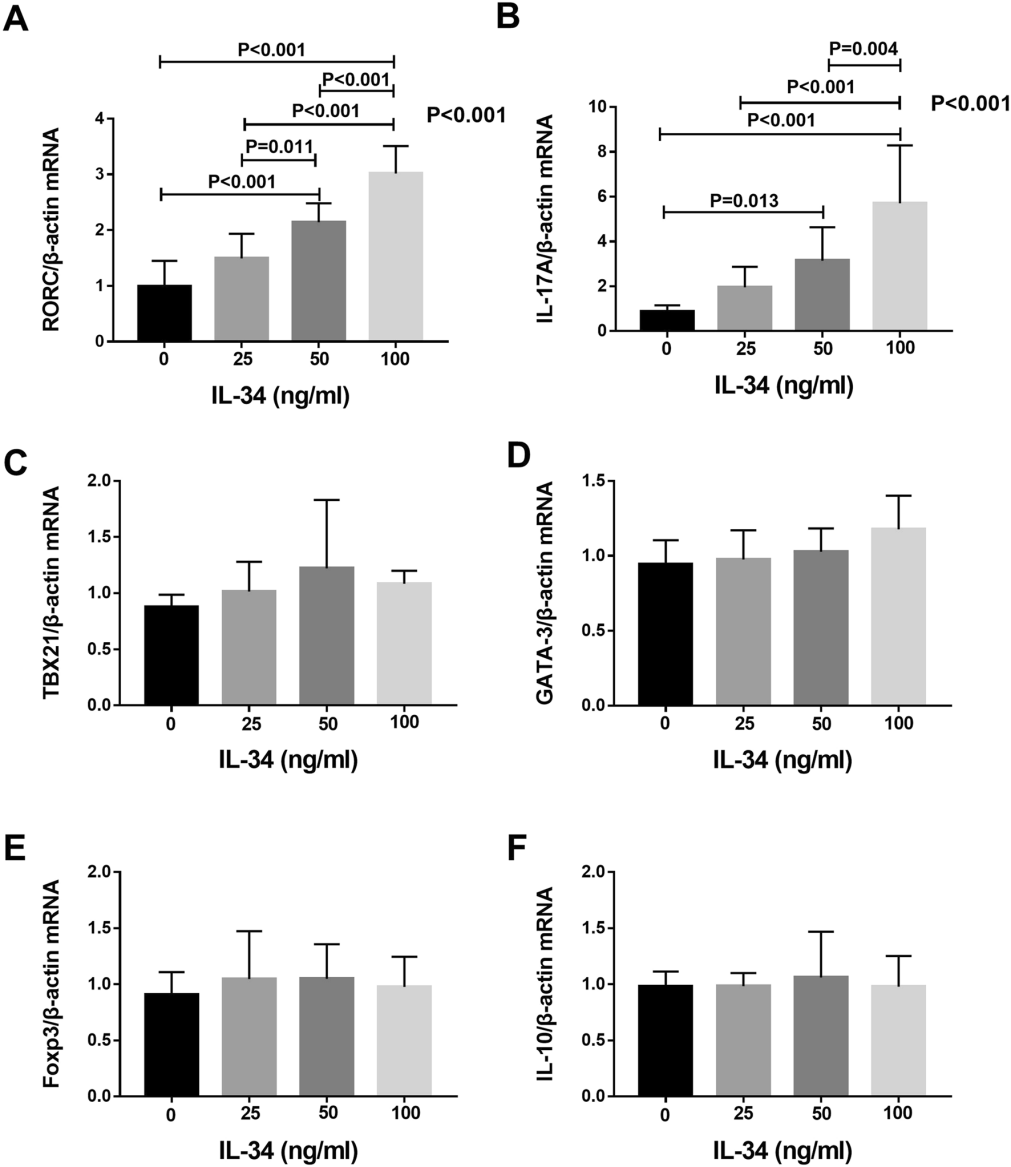
The differences in the gene expression levels of RORC (**A**), IL-17 (**B**), TBX21 (**C**), GATA3 (**D**), Foxp3 (**E**) and IL-10 (**F**) stimulated with different concentrations of IL-34 by reverse transcription-PCR analysis. PBMCs were stimulated with anti-CD3 and anti-CD28 for 3 days before RNA extraction. The differences between four groups were tested by ANOVA and after post Tukey test.

Through RT-PCR analysis, we found that IL-34 promoted the gene expression of RORC and IL-17 in PBMCs. For RORC, the RORC/β-actin mRNA relative expression values in the rhIL-34 (0, 25, 50, and 100 ng/ml) groups were 0.9905 ± 0.1450, 1.493 ± 0.1400, 2.142 ± 0.1077 and 3.014 ± 0.1563, respectively. There were statistically significant differences among the following group pairs: 0 ng/ml and 50 ng/ml (*P* < 0.001), 25 ng/ml and 50 ng/ml (*P* = 0.011), 25 ng/ml and 100 ng/ml (*P* < 0.001), 50 ng/ml and 100 ng/ml (*P* < 0.001), and 0 ng/ml and 100 ng/ml (*P* < 0.001). The effect on relative mRNA expression was dose dependent (Fig. 4A). We observed similar mRNA expression patterns for IL-17, which also exhibited mRNA upregulation by increasing concentrations of rhIL-34. For IL-17, the relative mRNA expression ratios of IL-17/β-actin in cells stimulated with rhIL-34 (0, 25, 50 and 100 ng/ml) were 0.8820 ± 0.0862, 1.957 ± 0.2878, 3.147 ± 0.4719 and 5.705 ± 0.816, respectively. There were significant differences between the 0 ng/ml group and 50 ng/ml group (*P* = 0.013), 0 ng/ml group and 100 ng/ml group (*P* < 0.001), 25 ng/ml group and 100 ng/ml group (*P* < 0.001), 50 ng/ml group and 100 ng/ml group (*P* = 0.004) (Fig. 4B). Furthermore, we detected the transcription factors and cytokines representing Th1, Th2 and Treg cells in PBMCs. However, we did not find that IL-34 could influence the transcription factor (TBX21, GATA-3 and Foxp3) or cytokine (IL-10) expression of these cells. Furthermore, there were no statistically significant differences among the groups (Fig. 4C–F).

### IL-34 promoted the secretion of IL-17A by PBMCs from RA patients

**Figure 5:**
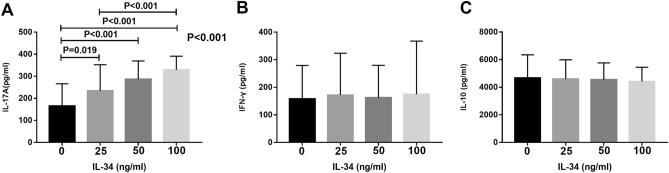
The differences between the IL-17 (**A**), IFN-γ (**B**) and IL-10 (**C**) expression stimulated with different concentrations of IL-34 by ELISA. Cell culture supernatants of PBMCs treated with different concentrations of IL-34 and anti-CD3 and anti-CD28 for 3 days were used. The differences between four groups were tested by ANOVA and after post Tukey test.

The levels of IL-17A, IFN-γ, IL-4 and IL-10 in the supernatants of PBMC cell cultures treated with different concentrations of IL-34 were detected by ELISA. IL-17A expression was significantly elevated by the stimulation with rhIL-34 (0, 25, 50 or 100 ng/ml) (165.1 ± 17.82 pg/ml, 234.2 ± 20.89 pg/ml, 286.7 ± 14.65 pg/ml and 329.1 ± 10.97 pg/ml, respectively). There were statistically significant differences between the following pairs of groups: 0 ng/ml and 25 ng/ml (*P* = 0.019), 0 ng/ml and 50 ng/ml (*P* < 0.001), 0 ng/ml and 100 ng/ml (*P* < 0.001), 25 ng/ml and 100 ng/ml (*P* < 0.001) (Fig. 5A). However, IL-34 had no significant impact on the production of IFN-γ or IL-10 (Fig. 5B,C). Moreover, the concentration of IL-4 was lower than the limit of detection of the ELISA kit. Thus, we did not perform any further analyses (data not shown).

## Discussion

In previous studies, IL-34 was shown to have a pro-inflammatory effect, and compared with those in healthy people, the levels of IL-34 in the serum and synovial fluid of RA patients were increased^17^. Our previous study showed that the serum levels of IL-34 in RA patients were significantly elevated and associated with disease activity, including the number of tender joints, ESR, CRP and DAS28-CRP^11^. In a pilot study, we showed that IL-34 might promote the secretion of IL-17 by PBMCs in RA patients^11^. These results showed that IL-34 might play a role in RA pathogenesis by regulating Th17 cells.

In our previous studies, we found that IL-34 promoted IL-17 production in PBMCs from RA patients but not in those from healthy donors^18^. Whether IL-34 can affect only the production of IL-17 in activated CD4 + T cells is still unclear. To confirm this effect, we investigated Th1 (IFN-γ), Th2 (IL-4), Th17 (IL-17) and Treg (IL-10) cells treated with increasing concentrations of rhIL-34. However, the exact effect of IL-34 on Th17 cells is not clear. Does IL-34 affect the differentiation and proliferation of Th17 cells or the expression of transcription factors involved inTh17 cell differentiation? Whether IL-34 has effects on the proliferation and function of other Th cell subtypes (Th1, Th2 and Treg subsets) has not been reported.

In this study, we found that IL-34 could stimulate the differentiation of Th17 cells in PBMCs from RA patients. However, IL-34 had no effect on the differentiation of other Th cell subsets, including the Th1, Th2 and Treg subsets. Moreover, we did not find the same effect of IL-34 on healthy controls (Supplementary Fig. 1A,B). These data suggested that the effect of IL-34 might be specific to RA.

Transcription factors are required for Th cell differentiation^19^. Our data showed that IL-34 led to activation of ROR-γt and thereby promoted the expression of IL-17. In contrast, IL-34 had no effect on the expression of transcription factors for Th1 (T-bet), Th2 (GATA-3) and Treg (Foxp3) cells. We further detected the mRNA expression of TBX21, GATA-3, RORC and FOXP3 by RT-PCR. We showed that IL-34 could increase the mRNA levels of RORC and IL-17. In contrast, IL-34 had no effect on the mRNA expression of TBX21, GATA-3 or FOXP3.

IL-17 is mainly produced by Th17 cells^20^. Our study showed that IL-34 promoted the secretion of IL-17 by PBMCs from RA patients in a dose-dependent manner. However, IL-34 had no effect on the secretion of IFN-γ or IL-10.

CSF-1R is the main receptor of IL-34^21^. A previous study showed that blocking CSF-1R in mice had a protective effect against collagen-induced arthritis and was more effective than a TNF-α antagonist^22^. Anti‐CSF‐1R therapy not only suppresses disease progression in a CIA mouse model but also significantly attenuates pro‐inflammatory cytokine production (TNF‐α, interferon‐γ, IL‐1β and IL‐6)^23^. Consistent with this finding, oral CSF‐1R inhibitors were shown to afford significant protection against bone erosion in mouse arthritis models^24^. Our future work may focus on blocking CSF-1R to learn more about the role of IL-34 in Th17 cell differentiation.

In conclusion, IL-34 can promote Th17 cell proliferation, transcription factor expression, and IL-17 secretion. We hypothesized that IL-34 acts upstream of Th17 cell differentiation and plays an important role in RA pathogenesis. This may provide a new target for RA therapy.

### Informed consent

We claim that all patients have been given informed consent prior to their inclusion in the study and the research was approved by Ethics Committee of the First Affiliated Hospital of China Medical University.

## Supplementary Information

Supplementary Figure 1. The frequency of Th17 cells from the Healthy control treated with different concentrations of IL-34. (A) Pseudo color plots show representative flow cytometric data of CD4+IL-17+T cells. (B) Bar charts show the frequency of Th17 cell..

Supplementary Information.
