# Supplementary figures and images for: Effect of IL-34 on T helper 17 cell proliferation and IL-17 secretion by peripheral blood mononuclear cells from rheumatoid arthritis patients
Source: Sci Rep. 2020 Dec 17;10:22239. doi: 10.1038/s41598-020-79312-z (PMC7746722; doi:10.1038/s41598-020-79312-z)

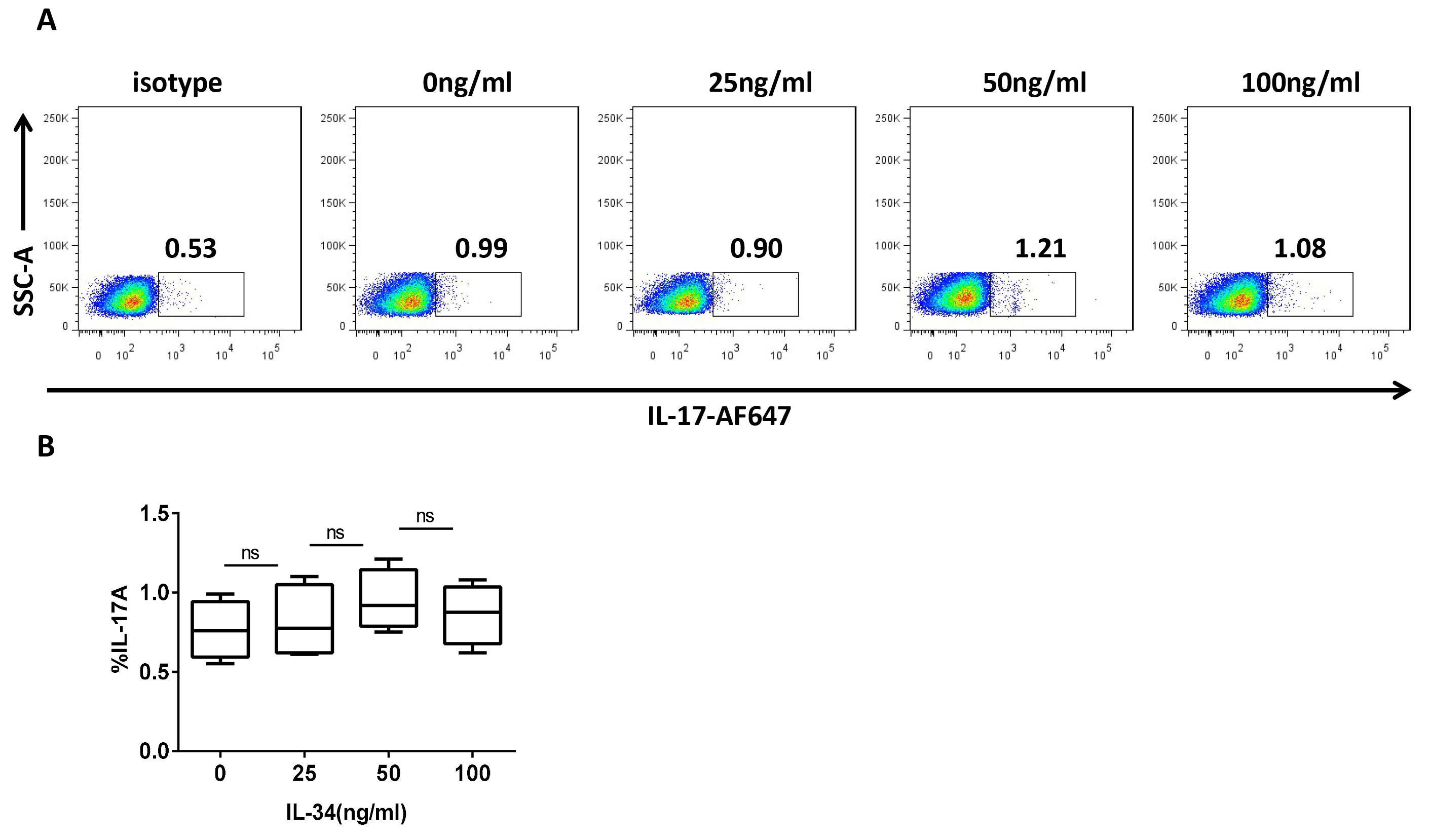

Supplement: Supplementary file 1 — Supplementary Figure 1. The frequency of Th17 cells from the Healthy control treated with different concentrations of IL-34. (A) Pseudo color plots show representative flow cytometric data of CD4+IL-17+T cells. (B) Bar charts show the frequency of Th17 cell.. [file 41598_2020_79312_MOESM1_ESM.tif]
