# Supplementary material for: Effect of IL-34 on T helper 17 cell proliferation and IL-17 secretion by peripheral blood mononuclear cells from rheumatoid arthritis patients
Source: Sci Rep. 2020 Dec 17;10:22239. doi: 10.1038/s41598-020-79312-z (PMC7746722; doi:10.1038/s41598-020-79312-z)
Supplement: Supplementary file 2 — Supplementary Information. [file 41598_2020_79312_MOESM2_ESM.pdf]

Supplementary information：

Effect of IL-34 on T helper 17 Cell Proliferation and IL-17 Secretion by Peripheral Blood Mononuclear Cells of Rheumatoid Arthritis Patients

Xin Li1,2*, Yi Meng Lei1*, Zi Yu Gao3, Bei Zhang1, Li Ping Xia1, Jing Lu1, Hui Shen1

*Xin Li and Yi Meng Lei contributed equally

1 Department of Rheumatology, 1st Hospital of China Medical University, Shen Yang, China, 110001

2 Department of Rheumatology, 1st Affiliated Hospital of Jin Zhou Medical University, Jin Zhou, China, 121000

3104k class 86, China Medical University, Shen Yang, China, 110001

Corresponding author: Shen Hui

Email:shenhuicam@sohu.com

Funding: This study was supported by a grant from the National Natural Science Foundation of China (No. 81373219) and a grant from the Liaoning Education Department (No.JC2019009).

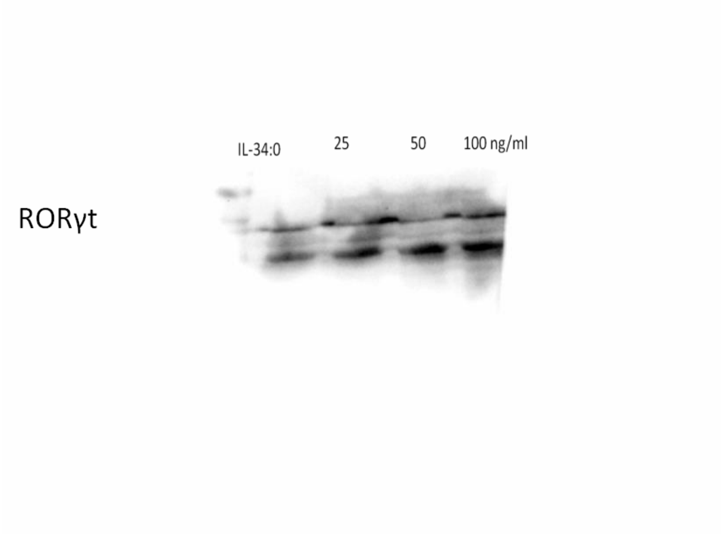
IL-34:0
25
50
100 ng/ml
RORγt

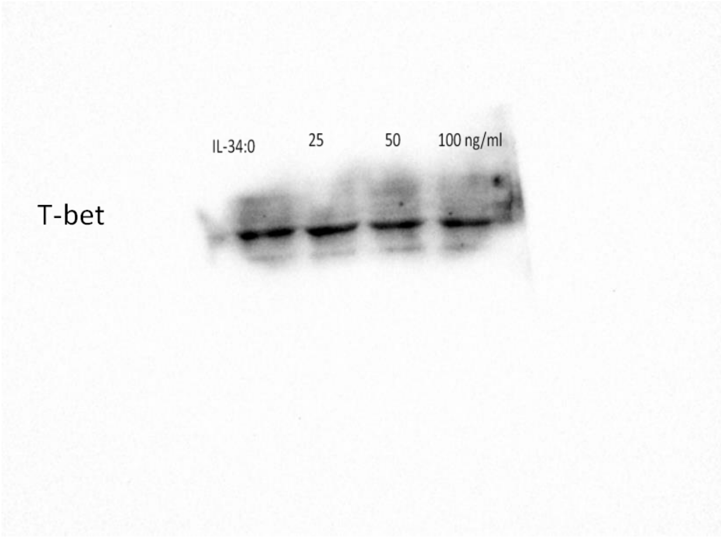
IL-34:0
25
50
100 ng/ml
T-bet

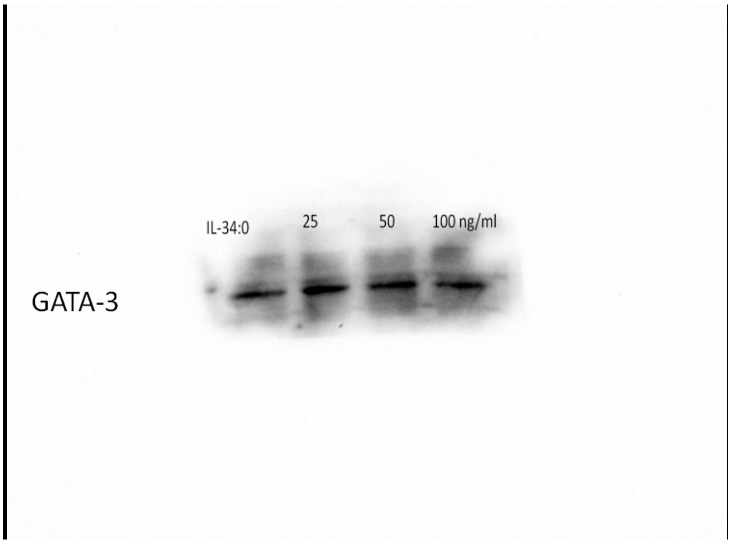
IL-34:0
25
50
100 ng/ml
GATA-3

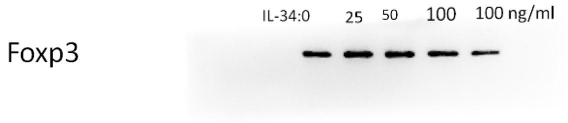
IL-34:0
25
50
100
100 ng/ml
Foxp3

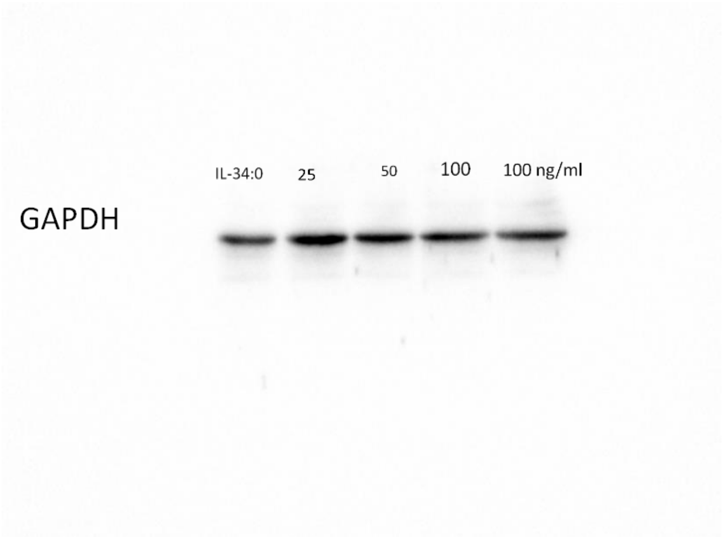
IL-34:0
25
50
100
100 ng/ml
GAPDH
